# Adult PTSD symptoms and substance use during Wave 1 of the COVID-19 pandemic

**DOI:** 10.1016/j.abrep.2021.100341

**Published:** 2021-03-03

**Authors:** Cheryl L. Currie

**Affiliations:** Faculty of Health Sciences, University of Lethbridge, M3083 Markin Hall, 4401 University Drive, Lethbridge, Alberta T1K 3M4, Canada

**Keywords:** COVID-19, Alcohol, Cannabis, PTSD, Gender, Socioeconomic status

## Abstract

**Introduction:**

This study examined associations between pandemic-related PTSD symptoms and substance use among adults, the role of gender and socioeconomic status in these outcomes, and the supports that adults needed to address these problems during Wave 1 of the COVID-19 pandemic in Alberta, Canada.

**Methods and Measures:**

Data were collected from 933 community-based adults without a previous diagnosis of PTSD in June 2020. The Primary Care PTSD Screen was adapted to assess pandemic-related PTSD symptoms. Participants were asked if alcohol or cannabis use had increased in the past month. Adjusted logistic regression models examined associations between pandemic-related PTSD symptoms and substance use.

**Results:**

More women (19%) than men (13%) met criteria for high pandemic-related PTSD symptomology, while a similar percentage (13.4% of women, 13.2% of men) reported significant increases in substance use during the pandemic. Adults 18–35 years; those who believed they would become infected with the virus; and those with low income, education, or pandemic-related job loss were more likely to report PTSD symptoms. High pandemic-related PTSD symptomology was associated with a significant substance use increase among both women (OR = 2.2) and men (OR = 2.3) in adjusted models. Many adults (50% of women, 40% of men) reported they needed help to address these problems.

**Conclusions:**

Pandemic-related PTSD symptoms were common among adults during Wave 1 of COVID-19. These symptoms were associated with a significant increase in substance use among women and men. Many adults voiced a need for help with these problems. Findings suggest substance use interventions that consider and address pandemic-related PTSD symptoms may be needed.

## Highlights

• About 13% of Alberta adults had significantly increased their substance use during the pandemic.
• These increases (alcohol and/or cannabis) did not differ by gender.
• Pandemic-related PTSD symptoms were associated with increased substance use.
• This association was moderate in strength and did not differ by gender.
• Almost 50% of adults indicated they needed supports to help them with these problems.

## 1 Introduction

The mental health and wellbeing of societies has been severely affected by the coronavirus disease 2019 (COVID-19) pandemic (United Nations, 2020). In Canada, about one quarter of adults reported poor to fair mental health in May 2020 compared to 8% in 2018, with similar changes reported in other countries (Codagnone et al., 2020, Statistics Canada, 2020a, Xiang et al., 2020). Given the global impact of COVID-19, these impacts likely go beyond increased symptomology to include clinically-significant changes in mental health status for some adults. Posttraumatic stress disorder (PTSD) has been reported in response to various epidemics and may be a particular concern during the COVID-19 pandemic (Boyraz & Legros, 2020, Lau et al., 2005, Liu et al., 2020). PTSD symptoms include intrusive recollection of a traumatic event, efforts to avoid stimuli associated with it, negative alterations in cognition or mood, and marked changes in arousal and reactivity (American Psychiatric Association, 2013). A review of emerging evidence found risk factors for increased PTSD symptomology during the pandemic include gender, levels of exposure, hospitalization, loss of a loved one, social isolation, and older age (Boyraz & Legros, 2020). A key rationale for this study was to examine if adults who were more vulnerable to the socioeconomic impacts of the pandemic were more likely to have pandemic-related PTSD symptoms. This examination would provide important information about vulnerable or at-risk groups for targeted mental health interventions (Blumenshine et al., 2008, Boyraz & Legros, 2020, Quinn and Kumar, 2014).

The second rationale was to examine associations between pandemic-related PTSD symptoms and substance use by gender. It is well documented that persons who are isolated and stressed often turn to substances to alleviate negative feelings (Cooper et al., 1995, Volkow, 2020). This is a concern during a pandemic given alcohol and drugs weaken the body’s immune system and ability to cope with infectious disease (Eisenstein et al., 2008, Friedman et al., 2006). Early reports suggest substance use has increased more among women than men during the pandemic; suggesting gender-stratified analyses are needed (Biddle et al., 2020, Lu et al., 2020). In Australia, 23% of women and 18% of men who consume alcohol have reported increased use during the pandemic (Biddle et al., 2020). Common reasons for increased alcohol use among Australian adults were spending more time at home and boredom. However, women were more likely than men to cite stress, anxiety, and worry about COVID-19 as reasons why their alcohol use had increased during the pandemic (Biddle et al., 2020). Building on these findings, a third rationale for this third study was to examine whether pandemic-related PTSD symptoms were statistically associated with increased substance use separately among Canadian women and men during the pandemic. Finally, to inform the resourcing of interventions, this study sought to understand the proportion of population-based adults in Alberta who believed they personally needed supports to address their substance use, stress levels, and/or mental health during the pandemic.

In summary, this study sought to: (1) Examine gender and socioeconomic differences in pandemic-related PTSD symptoms and substance use among adults; (2) examine associations between PTSD symptoms and substance use among adults using gender-stratified models; and (3) understand the supports community-based adults were seeking to address their substance use, mental health, or stress levels during the pandemic.

## 2 Materials and methods

### 2.1 Study design and eligibility criteria

This cross-sectional study was derived from Canada’s largest online panel with 400,000 members (Opinion, 2019). The panel is demographically representative of the Canadian adult population as measured by the most recent Census. Data collection was limited to a single province to promote consistency in exposure to COVID-19 and containment measures. Eligible participants were adults living in non-institutionalized private dwellings in Alberta in June 2020 who had not been diagnosed with PTSD before the pandemic. The study was approved by the University of Lethbridge Human Research Ethics Committee (ID 2020–054). Informed consent was obtained from all participants.

### 2.2 Sample and procedure

Data were collected over a 7-day window in June 2020. On June 1, 2020 Leger Opinion emailed a study invitation to adults who had previously volunteered for its online panel and lived in Alberta, Canada. The 15-minute survey was open until approximately 1,000 adults completed it on June 7, 2020 (*N* = 1,025). The final sample was 933 adults after those with a pre-pandemic PTSD diagnosis (*n* = 49, 5.0%) or missing data (*n* = 43, 4.2% of sample) were removed.

### 2.3 COVID-19 context

Participants answered questions about PTSD symptoms and substance use in the past 4 weeks (i.e., May 1 to June 7, 2020 depending on the date of survey completion). Societal restrictions to prevent COVID-19 had been in place for 6 weeks by May 1 in Alberta. Stores that sold alcohol and cannabis were deemed essential services and remained open throughout the pandemic without interruption. In mid-May, Phase 1 of a staged relaunch brought gradual reopening of businesses deemed nonessential as well as restaurants, daycares, and playgrounds (Government of Alberta, 2020b). Between May 1 - June 7, 2020 most adults continued to work from home, K to 12 and post-secondary education took place online across the province, and the US border remained closed. New COVID-19 infections had climbed through March and April, peaking on April 30, 2020 at 2,992 cases, or 68 active cases per 100,000 population in Alberta (Government of Alberta, 2020a). During this time, the COVID-19 hospitalization rate was 4.5%; and the case fatality ratio was 2.0% (Government of Alberta, 2020a). A precipitous decline in new infections followed, with less than 400 active cases by the first week of June when the present data were collected (<9 active cases per 100,000 population) (Government of Alberta, 2020a). While many Canadians lost jobs due to COVID-19, in April 2020 the federal government began providing those unemployed for any reason with $2,000 per month to ease the financial impacts of the pandemic on the population (Goverrment of Canada, 2020).

### 2.4 Measures

#### 2.4.1 PTSD

Participants were asked if they had received a PTSD diagnosis before the pandemic began. Those who had received a formal diagnosis were excluded from the analysis. The 5-item Primary Care PTSD Screen for DSM-5 was adapted to assess assesses five pandemic-related symptoms of PTSD occurring in the past month by substituting the words “the event” with “COVID-19” in the six places it occurred in the original scale (Prins et al., 2016). For example, the first question was modified to: “*In the past month, have you had nightmares about COVID-19 or thought about COVID-19 when you did not want to?*” Response options were consistent with the original scale (1 = yes, 0 = no). Internal consistency in the present sample was good (Cronbach’s α = 0.73). Validation research suggests a cut-point of 3 (i.e., 3 or greater) is optimally sensitive for high PTSD symptomology (*r* = 0.78), and was used to differentiate those with high and low pandemic-related PTSD symptomology in this study (Prins et al., 2016).

#### 2.4.2 Substance use

Two questions asked adults if they had used alcohol or cannabis in the past month (yes or no). Those who had were asked if past-month alcohol and cannabis use had increased very much, decreased very much, or stayed about the same. Participants who indicated their use of alcohol or cannabis had increased very much were coded as *1 = substance use increased very much (*i.e.*, significantly) in the past month*. Those who reported no change in use, a decrease in use, or that they did not consume alcohol or use cannabis in that timeframe were coded as *0 = substance use did not increase in the past month*. Substance use changes were examined with abstainers included given cross-sectional studies examine prevalence, and the defined population in a prevalence estimate includes those at risk and those not at risk for the target behavior (Szklo & Nieto, 2019). Calculations that exclude abstainers produce inflated estimates, do not represent the true frequency of behaviors in a population, and have limited generalizability (Lange, Probst, Rehm, & Popova, 2017).

#### 2.4.3 Supports needed

Three questions asked participants if they wanted to make changes in their stress levels (yes or no), mental health (yes or no) or substance use (yes or no) in preparation for possible COVID-19 infection. Adults who indicated they wanted to make one or more of these changes (*n* = 462) were asked: “*What supports do you need to make this change?*” Participants were presented with a list of options (select all that apply) including support from friends, a family doctor, a psychological counsellor, a health coach, a spiritual or religion mentor, other, and no supports/not sure.

#### 2.4.4 Covariates

Age categories, gender, education, marital status, and income group were collected. Income group was collected as cost of living varies widely across Alberta, making income in dollar amounts less useful. Participants were asked if they had lost their job/been laid off due to COVID-19 (yes or no), if they had contracted COVID-19, and if they believed they would contract COVID-19 in the next year (yes, unsure, no).

### 2.5 Sample size calculation

The required sample size was calculated based on data that found 29% of adults who met criteria for high PTSD symptomology after the 2004 Florida hurricane reported increased alcohol use compared to 6% exposed to the hurricane who did not develop high PTSD symptomology (Fullerton et al., 2013). Using this information, the present sample size was estimated assuming 25% of adults with high pandemic-related PTSD symptoms would report increased substance use compared to 5% of adults exposed to the pandemic who did not develop high PTSD symptomology. Using a chi-squared statistic and assuming a gender-stratified analysis, 59 women and 59 men with high PTSD symptomology, and 59 women and 59 men with increased substance use were needed to achieve 80% power at α (two-sided) = 0.05 (Browner, Newman, & Hulley, 2013). In the present study, 89 women and 60 men had high PTSD symptomology, while 63 women and 61 men increased their substance use. Given 11% of adults experienced mental health problems and/or substance use increases after the hurricane, a sample of 1,000 adults was deemed sufficient to achieve adequate power (Fullerton et al., 2013). In the present study, 25% of adults experienced high PTSD symptomology and/or had increased their substance use very much during the pandemic.

### 2.6 Statistical approach

Crosstabs and odds ratios with 95% confidence intervals (CIs) examined differences in PTSD symptoms and increased substance use by gender and socioeconomic variables (income group, education, job loss). Gender-stratified logistic regression models and 95% CIs assessed the odds of increased substance use among adults with high PTSD symptomology, adjusting for demographic and socioeconomic variables (age, marital status, education, income group, and job loss due to COVID-19). The p-value was set at 0.05. Missing data were handled using listwise deletion. Analyses were run using SPSS 27.0.

## 3 Results

Participants ranged in age from 18 to 85 years. Approximately two-thirds were married or living common law and had completed post-secondary training (Table 1). The proportion of female participants (51%) matched population estimates (Statistics Canada, 2017). Approximately 15% had lost their job or had been permanently laid off due to the pandemic. The percentage unemployed was high (13.3%) but consistent with an elevated Canadian unemployment rate of 13.7% during this time period due to the pandemic (Statistics Canada, 2020b). No participants had been diagnosed with COVID-19 in this study. Six in ten adults believed or were unsure if they would contract COVID-19 in the next 12 months.

**Table 1:** Sample characteristic.

Sample Characteristics	*N* (%)	High PTSD Symptom Subgroup, *n* (%)	Unadjusted OR (95% CI)
Total sample	933 (100)	144 (100)	
Gender			
Women	471 (50.5)	87 (18.6)	**1.50 (1.10, 2.04)**
Men	462 (49.5)	57 (12.4)	1.0 (Reference)
Age			
18–34	229 (24.5)	61 (26.9)	**3.38 (2.10, 5.45)**
35–54	398 (42.7)	53 (13.4)	1.42 (0.88, 2.29)
55+	306 (32.8)	30 (9.8)	1.0 (Reference)
Education			
≤University of college degree	323 (34.6)	60 (18.7)	1.35 (1.00, 1.83)
University/college degree	610 (65.4)	84 (13.8)	1.0 (Reference)
Marital status			
Single and never married	188 (20.2)	45 (24.1)	**2.15 (1.43, 3.24)**
Single and previously married	126 (13.5)	20 (15.9)	1.28 (0.75, 2.18)
Married/living common law	618 (66.3)	79 (12.8)	1.0 (Reference)
Household income			
Low or low-middle income	276 (29.6)	58 (21.2)	**1.88 (1.14, 3.09)**
Middle income	441 (47.3)	59 (13.4)	1.09 (0.67, 1.77)
Upper-middle or upper income	216 (23.2)	27 (12.5)	1.0 (Reference)
Job loss due to COVID-19			
Yes	141 (15.1)	31 (22.0)	**1.68 (1.08, 2.63)**
No	792 (84.9)	113 (14.3)	1.0 (Reference)
Have you had COVID-19			
Unsure (had symptoms, but not tested)	125 (13.4)	28 (22.6)	**1.73 (1.09, 2.75)**
No	806 (86.6)	116 (14.4)	1.0 (Reference)
Will you contract COVID-19 in next year?			
Yes	106 (11.4)	31 (29.4)	**3.24 (1.91, 5.49)**
Unsure	452 (48.4)	70 (15.5)	1.44 (0.96, 2.17)
No	373 (40.0)	42 (11.3)	1.0 (Reference)

### 3.1 Pandemic-related PTSD symptoms

The average pandemic-related PTSD score was 1.0 (SD = 1.4, range 0 to 5), with 47.8% of adults reporting at least one of five PTSD symptoms in the past month. Overall, 15.4% of the sample (*n* = 144 adults) met criteria for high pandemic-related PTSD symptomology, defined by scores of 3 or more. As shown in Table 1, the strongest correlates for high pandemic-related PTSD symptoms was young age, followed by the belief that one would contract COVID-19 in the next year. Specifically, adults aged 18–34 years had more than a three-fold increase in the odds of reporting high pandemic-related PTSD symptomology compared to adults aged 56 years and older. Adults who believed they would contract COVID-19 in the next year also had more than a three-fold in increase in the odds of high pandemic-related PTSD symptomology than adults who believed they would not become infected. These associations were moderate in strength (i.e., odds ratios above 2 and below 5) (Webb, Bain, & Page, 2020). A number of other socioeconomic variables were associated with high pandemic-related PTSD symptoms including low/low-middle income, job loss due to the pandemic, non-married status, and female gender. These associations were typically weak in strength (i.e., odds ratios <2.0).

### 3.2 Gender and pandemic-related PTSD symptoms

Table 2 provides detail on differences in pandemic-related PTSD symptomology between women and men. Women had a pandemic-related PTSD score that was significantly higher than men (*independent samples t-test* = 3.63, *df* = 920, *p* = 0.001). As well, approximately 19% of women met criteria for high pandemic-related PTSD symptomology compared to approximately 13% of men; this difference was statistically significant (*Pearson’s Chi Square* = 6.50, *df* = 1, *p* = 0.01)

**Table 2:** Gender-stratified frequency of PTSD symptoms in the full sample, and among those who reported increased substance use in the past month.

	Full Sample			Subsample in Which Substance Use Increased		
In the Past Month:	Women *n* = 467	Men *n* = 464	*p* value	Women *n* = 63	Men *n* = 61	*p* value
1. Have you had nightmares about COVID-19 or thought about COVID-19 when you did not want to?	17.8%*	8.8%	0.001	33.3%*	14.8%	0.02
2. Have you tried hard not to think about COVID-19, or went out of your way to avoid being reminded of COVID-19?	32.4%*	24.2%	0.005	42.9%	43.3%	0.96
3. Have you been constantly on guard, watchful, or easily startled?	24.8%	23.4%	0.62	39.7%	36.1%	0.68
4. Have you felt numb or detached from people, activities or your surroundings?	30.5%*	23.3%	0.01	46.8%	49.2%	0.79
5. Have you felt guilty or been unable to stop blaming yourself or others for COVID-19 or problems created by COVID-19?	10.9%*	6.0%	0.008	20.6%	11.5%	0.17
Mean PTSD score (SD)	1.2* (1.4)	0.9 (1.2)	0.001	1.9 (1.7)	1.6 (1.4)	0.10

* Statistically significant chi-square test.

In Table 2, the full sample is stratified by gender and examined by PTSD symptom. Women were significantly more likely than men to report COVID-19 nightmares and intrusive thoughts; trying hard to avoid thinking about or being reminded of the pandemic; feeling numb or detached from people, activities or their surroundings; and feeling guilty or unable to stop blaming themselves or others for the pandemic.

### 3.3 Increased substance use

Significant increases in past month alcohol use were reported by 12.8% of women and 10.7% of men. Significant increases in past month cannabis use were reported by 2.0% of women and 4.1% of men. Taken together, 13.4% of women and 13.2% of men reported their substance (alcohol and/or cannabis use) use had increased significantly in the past month.

### 3.4 Pandemic-related PTSD symptoms and substance use

In Table 2, the subsample that had increased their substance use “very much” in the past month is examined by PTSD symptom, stratified by gender. Overall, adults who had increased their substance use reported more pandemic-related PTSD symptoms. For example, approximately 43% of men who had increased their substance use reported they were trying hard not think about or be reminded of COVID-19 compared to 24% of men who had not increased their substance use. Among women, a third who had increased their substance use were experiencing COVID-19 nightmares and intrusive thoughts compared to 18% of women who had not increased their substance use.

As shown in Table 3, high pandemic-related PTSD symptoms were associated with more than a two-fold increase in the odds of increased substance use in the past month among both women and men. The strength of these associations changed little after adjustment for age, marital status, SES, and job loss due to COVID-19.

**Table 3:** Unadjusted and adjusted odds ratios (ORs) for increased past-month substance use among adults with high vs. low pandemic-related PTSD symptoms.

Pandemic-Related PTSD Symptoms	Increased Substance Use: OR (95% CI)		Increased Substance Use: AOR (95% CI)^a^	
	Women	Men	Women	Men
High (≥3)	2.58 (1.43, 4.63)	2.73 (1.41, 5.30)	2.22 (1.20, 4.10)	2.30 (1.13, 4.66)
Low (0–2)	1.0 (Reference)	1.0 (Reference)	1.0 (Reference)	1.0 (Reference)

a ORs adjusted for age, marital status, education, income, and job loss due to COVID-19.

### 3.5 Needed supports

Significantly more women (52.0%) than men (41.1%) wanted to make changes in their stress levels, mental health or substance use in preparation for possible COVID-19 infection (*Chi-Square test* = 9.60, *df* = 3, *p* = 0.02). About a third of all adults (34.3%) wanted to reduce stress, about one quarter (27.3%) wanted to improve their mental health, and about one in ten (8.9%) wanted to reduce substance use. Adults who indicated they wanted to make one or more of these changes (*n* = 462) were asked about the supports they needed to make the changes and presented with a list of options.

**Fig. 1:**
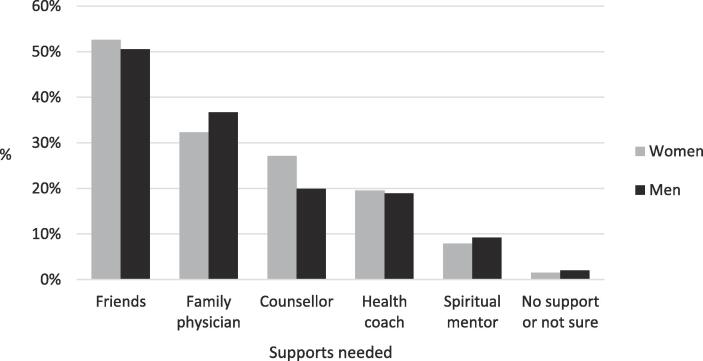
Supports needed by community-based adults who want to make changes in their stress, mental health, or substance use during the COVID-19 pandemic (*N* = 462).

As shown in Fig. 1, few who wanted to make changes believed they did not need support or were unsure of the supports they needed. The most common supports needed to improve stress, mental health, or reduce substance use was support from friends, followed by support from a family physician, a psychological counsellor, and a health coach. A third of the sample (33.3%) selected more than one support. There were no statistically significant gender differences in the supports that adults needed to address their stress, mental health, or substance use during the pandemic.

## 4 Discussion

Data collected for this study represent a one-month period when the burden that the COVID-19 pandemic had placed on a community-based sample of adults was lifting for the first time after a first two-month wave of increasing infections and containment measures. A significant proportion of women (19%) and men (13%) were experiencing pandemic-related PTSD symptoms during this period despite having no previous diagnostic history of the disorder.

In contrast to a recent review, older age was not a risk for increased PTSD symptoms in this study (Boyraz & Legros, 2020). Young age (18–34 years) was the strongest correlate for pandemic-related PTSD symptoms and associated more than a three-fold increase in high PTSD symptomology. Belief that one would become infected with COVID-19 was the second strongest risk factor for high pandemic-related PTSD symptoms. Those who were socioeconomically disadvantaged due to low income, lack of a post-secondary qualification, or pandemic-related job loss were also more vulnerable to high pandemic-related PTSD symptoms, despite a federal emergency response benefit of $2,000 per month provided to all unemployed Canadians during this period. Women had higher pandemic-related PTSD scores than men, and were particularly more likely to be experiencing COVID-19 nightmares and intrusive thoughts.

In contrast to Australian data, the present findings do not suggest a gender difference in substance use changes during the pandemic, with approximately 13% of women and men reporting significant increases in their substance use (alcohol and/or cannabis) in the past month in this Canadian sample (Biddle et al., 2020). High pandemic-related PTSD symptoms were associated with an approximate two-fold rise in the odds of increased substance use in the past month. The strength of this association was statistically significant and similar for women and men. Approximately 43% of women and men who had increased their substance use reported they were trying hard not to think about or be reminded of the pandemic. Similarly, almost 40% of women and men who had increased their substance use were feeling constantly on guard, watchful, or easily startled in the past month; and approximately half were feeling numb or detached from people, activities, or their surroundings. A hypothesis that could be put forward from these results is that adults had increased their substance use to cope with these pandemic-related PTSD symptoms, given the use of substances to avoid and escape negative thoughts, emotions, and physiologic stress reactions is widely recognized in the literature (Cooper et al., 1995, Currie et al., 2015).

About 50% of women and 40% of men wanted to make changes in their stress levels, mental health, or substance use in preparation for possible COVID-19 infection. Almost all indicated they needed support to do so, particularly from friends, physicians, counsellors, and/or health coaches.

### 4.1 Limitations

Study limitations include use of a cross-sectional design which precludes inferences about temporal sequence and causation. A convenience panel sample was used. This sample was representative of the general Canadian population; however, healthy volunteer bias and residual confounding remain concerns. The measure used to examine PTSD is a screen that examines symptoms; not a PTSD diagnosis. It is possible that some PTSD symptoms were unrelated to the pandemic despite adapting the screen to refer to COVID-19 and the removal of those with a pre-pandemic PTSD diagnosis from the sample. Recall bias and underreporting is possible given substance use is a socially undesirable behavior.

### 4.2 Conclusions

This study examined adults who had just experienced two months of increasing COVID-19 cases and containment measures. Findings suggest pandemic-related PTSD symptoms were common. These symptoms were associated with a significant increase in substance use during this period. Many adults voiced a need for help with these problems. Findings suggest substance use interventions that consider and address pandemic-related PTSD symptoms may be needed.

## Funding details

This study was funded by Alberta Innovates Translational Health Research Chair Award awarded to CL Currie (#201300491).

## Data availability statement

Data is available from the corresponding author on reasonable request.

## Ethical approval

All procedures performed in studies involving human participants were in accordance with the ethical standards of the institutional and/or national research committee (University of Lethbridge Human Research Ethics Committee ID 2020-054) and with the 1964 Helsinki declaration and its later amendments or comparable ethical standards.

## CRediT authorship contribution statement

**Cheryl L. Currie:** Conceptualization, Formal analysis, Funding acquisition, Methodology, Project administration, Writing - original draft, Writing - review & editing.
